# Dentists and Anæsthesia

**Published:** 1909-05-01

**Authors:** 


### Dentists and Anæsthesia.

As had been foreseen by those who have studied the Bill to regulate the administration of anæsthetics, which was read a first time in the House of Commons on March 25, the clauses by which it is proposed to deprive dentists, as well as other unqualified persons, of the privilege of administering all general anæsthetics are exciting the active opposition of the dental profession. In a circular letter to the members of the British Dental Association the Secretary of that body, on behalf of the executive, brings forward several arguments against the Bill as it at present stands. We have already (The Hospital, March 20, 1909, p. 629) discussed by anticipation most of the provisions to which dentists object, and have pointed out that there is a good deal to be said on both sides of the question; we trust, therefore, that dental surgeons will not regard it in any way as a sign of hostility to some of their contentions if we mention one or two weak points in others. Their Association asserts that the average dentist has more experience and skill in the use of anæsthetics for dental purposes than the average medical man. This may possibly be true so far as nitrous oxide is concerned; but it certainly does not apply to the new generation of medical men who have received adequate instruction in this branch of practice, and in a few years' time it will probably no longer hold good. More than that, the dental profession must allow that the more skilful and successful a dentist is, the more he insists on employing none other than a qualified medical anæsthetist. The meaning of this very significant fact is that the best class of dentists do not consider themselves on an average as competent to administer general anæsthetics as the average medical practitioner of a corresponding status. And if that is what the conduct of the best class of dentists means, it stands to reason that the proficiency of less flourishing dental surgeons in anæsthetic work is at least not ideal.

Nor can much weight be attached to a statement that "the administration of anæsthetics is an integral part of the practice of dentistry." This is a somewhat cryptic pronouncement, the validity of which depends entirely on what is read into it. That dentistry without anæsthesia would not be modern scientific dentistry at all is true: the same is very much more true of operative surgery. But to argue on that basis that a dental surgeon is well advised to anæsthetise his patient and then extract his tooth would be as foolish as to suggest that a general surgeon can equally afford to dispense with an anæsthetist. This is, after all, the main crux of the question; whether or no dentists are to be allowed to go on extracting under gas which they have themselves administered. There can be no doubt that the system is vicious, and ought, if possible, to be abolished; there is also no doubt that the anæsthesia obtained by those dentists who follow this practice is very often most imperfect. On the other hand, it is urged that statistics of deaths under gas administered thus do not bear out the view that the danger is of any but the most minute dimensions; and it must be admitted that very few such deaths indeed are on record compared with those from the major anæsthetics used in surgical practice. Lastly, the Dental Association holds that the omission of local anæsthesia is calculated to defeat the object of the Bill. For the prohibition of general anæsthetics to dentists would merely lead to a greater extension of the practice of using local anæsthesia. On general principles we think the Association is here most certainly in the right. Moreover, whether dentists are or are not to be made exceptions, the non-medical public should certainly be prohibited from the use of dangerous drugs for producing local anæsthesia. To the argument that the Bill will make extraction under gas too costly for large sections of the community we have already given prominence. This is probably one of the strongest parts of the Dental Association's case; and it is a consideration of much moment. As not infrequently happens, the dictates of scientific perfection and of material practicability seem to be for the time being irreconcilable. To anyone who can show how to satisfy fully the demands of both, dentists and doctors will both owe a debt of gratitude.

